# Enhancing interoceptive awareness in community-dwelling older adults: effects of a psychomotor intervention mediated by creative dance

**DOI:** 10.3389/fpsyg.2025.1515393

**Published:** 2025-08-18

**Authors:** Hugo Rosado, Patrícia Motta, Ana Cruz-Ferreira, Catarina Pereira

**Affiliations:** ^1^Departamento de Desporto e Saúde, Escola de Saúde e Desenvolvimento Humano, Universidade de Évora, Évora, Portugal; ^2^Comprehensive Health Research Centre (CHRC), Universidade de Évora, Évora, Portugal

**Keywords:** aging, body awareness, emotional regulation, interoception, music

## Abstract

**Objective:**

This study aimed to investigate the effects of a psychomotor intervention mediated by creative dance on interoceptive awareness in community-dwelling older adults.

**Methods:**

This 12-week non-randomized clinical trial involved 34 participants (74.6 ± 6.6 years), divided into two groups. The experimental group (EG) engaged in a psychomotor intervention (3×/week; 60 min/session), while the control group (CG) continued their usual daily activities. Interoceptive awareness was assessed using the Multidimensional Assessment of Interoceptive Awareness at baseline and post-intervention.

**Results:**

Within-group comparison showed significant improvements in the EG for the scales of Noticing, Not-Worrying, Attention Regulation, Emotional Awareness, Self-Regulation, and Trusting, (*p* < 0.05). Post-intervention comparison between groups revealed significant differences in all aforementioned scales (*p* < 0.05), except for Not-Worrying. Overall, the CG maintained or decreased their results. The improvements observed in the EG were clinically relevant, with effect sizes ranging from medium to large.

**Conclusion:**

Our study results highlight the potential of a psychomotor intervention mediated by creative dance for enhancing interoceptive awareness in community-dwelling older adults, contributing to better emotional regulation and overall well-being. This effective intervention can be a valuable strategy for promoting healthy aging.

## 1 Introduction

Life expectancy continues to increase worldwide (Eurostat, 2022). While this is a positive development, aging is accompanied by physiological, sensorimotor, and emotional changes that can impact overall well-being (Eurostat, 2020). Emotional changes are particularly relevant, as effective emotional regulation in older adults is important for successful aging (Ulus and Aisenberg-Shafran, 2022).

Interoception plays a key role in emotional regulation, as it involves recognizing internal bodily signals that often reflect emotional states (e.g., breathing, muscle tension, or heart rate) (Mehling et al., 2018). These sensations are often the first indicators of emotional states, and being able to recognize them supports better self-regulation (Hanley et al., 2017). In the context of aging, this becomes especially relevant since older adults often experience a decline in interoceptive awareness (Murphy et al., 2018), which may contribute to reduced self-regulation, emotional awareness, and psychological well-being (Ulus and Aisenberg-Shafran, 2022). Enhancing interoceptive awareness may therefore support emotional stability and promote healthier aging.

Additionally, age-related changes often result in loss of independence, whereby policies and strategies engaging in physical activity to promote healthy aging are needed (Szychowska and Drygas, 2022). Psychomotor interventions, particularly those mediated by dance, may offer a promising approach to enhancing interoceptive awareness in older adults (Lebre et al., 2020). A psychomotor intervention designed for community dwellings uses the body and movement as mediators to promote sensorimotor, neurocognitive, and affective-emotional abilities (Rosado et al., 2022).

Among the expressive modalities that can be incorporated into psychomotor interventions, creative dance is particularly promising for enhancing interoceptive awareness, given its holistic and integrative use of movement, rhythm, and attention to internal body sensations (Giromini and Lesage, 2015; Guzman et al., 2016). This is especially relevant for older adults, who often experience age-related interoceptive decline (Murphy et al., 2018). Beyond physical improvements, creative dance fosters emotional expression and subjective well-being through spontaneous movement (Cruz-Ferreira et al., 2015; Martin-Wylie et al., 2024), and tends to promote adherence due to its enjoyable and socially engaging nature (Fong Yan et al., 2018).

However, despite some studies emphasizing the benefits of creative dance in promoting healthy aging (Cruz-Ferreira et al., 2015; Joung and Lee, 2019), its effects on interoceptive awareness in older adults remain largely unexplored. This study addresses that gap by applying an expressive, body-based approach and assessing its impact using a validated multidimensional tool. In this context, this study aimed to investigate the effects of a psychomotor intervention mediated by creative dance on interoceptive awareness in community-dwelling older adults.

## 2 Methods

### 2.1 Study design and participants

This study is a 12-week single-blinded non-randomized clinical trial (NRCT) with a quasi-experimental design. Conducted between March 2019 and July 2020, the study followed the SPIRIT 2013 checklist (https://www.spirit-statement.org/publications-downloads/). The protocol was retrospectively registered at ClinicalTrials.gov (ID: NCT04311931).

Participants were assigned by convenience to either the experimental group (EG: attended a psychomotor intervention mediated by creative dance) or the control group (CG: maintained their daily activities). During the study period, their routines mainly consisted of domestic tasks, occasional walking, and passive social activities (e.g., community lunches). Regular verbal reports indicated that participants in the CG did not engage in structured physical activity, dance classes, or similar interventions. At the end of the study, those in the CG were offered a similar intervention.

The study enrolled 37 community-dwelling older adults from Portugal. Recruitment strategies included flyers, presentations (e.g., senior university; recreational centers), and word of mouth. The inclusion criteria were: age ≥ 60 years; absence of cognitive impairment as measured by the Clock Drawing Test (score > 18 points) (Mendez et al., 1992); independent mobility; and no participation in any dance program within the last 12 months. Three of the 37 eligible volunteers were excluded, and the remaining 34 participants were allocated into the EG (15 females, 2 males) and the CG (16 females, 1 male). All participants completed the study and provided written informed consent.

### 2.2 Rationale for non-randomized clinical trial

A NRCT was performed due to logistical constraints inherent to community-based interventions. Participants were allocated to both groups based on their availability and location, which helped ensure adherence and feasibility. Although randomization was not feasible, the groups were matched at baseline on key variables, including age, gender and education.

### 2.3 Procedures

Assessments were conducted at baseline and after 12 weeks of intervention. Participants were individually assessed by the same trained evaluator, who holds a degree in Psychomotor Rehabilitation. The evaluator was blinded to both the study’s objectives and the participants’ group allocation. All assessments were performed in a quiet room (at the university laboratory).

### 2.4 Outcome measures

Interoceptive awareness: self-reported interoceptive awareness was assessed by the Multidimensional Assessment of Interoceptive Awareness (MAIA) (Mehling et al., 2018). The adapted and validated Portuguese version of the MAIA was used (Machorrinho et al., 2019). This version included 33 items and seven scales, namely Noticing (three items: awareness of uncomfortable, pleasant, and neutral body sensations), Not-Distracting (four items: tendency to self-distract or not from body sensations of pain or discomfort), Not-Worrying (four items: ability to maintain emotional stability when experiencing sensations of pain or discomfort), Attention Regulation (seven items: ability to control attention to body sensations), Emotional Awareness (five items: understanding the connection between body sensations and emotional states), Self-Regulation (seven items: capacity to manage distress by focusing on body sensations), and Trusting (three items: perceiving one’s body as safe). Each item ranged from 0 (never) to 5 (always) points.

#### 2.4.1 Complementary outcomes measures

The sociodemographic characteristics were assessed through a scripted interview-based questionnaire. Cognitive function was assessed using the Clock Drawing Test (CDT), a widely recognized tool for detecting cognitive impairments in otherwise healthy older adults (Aprahamian et al., 2009). The CDT followed the methodology proposed by Mendez and colleagues (Mendez et al., 1992), with scores ranging from 0 (worst) to 20 (best) points. Participants’ body weight (kg) and height (m) were measured using a calibrated scale (Seca 760, Hamburg, Germany) and a stadiometer (Seca 206, Hamburg, Germany), respectively. Body mass index (BMI) was calculated using the formula (kg/m^2^).

### 2.5 Psychomotor intervention mediated by creative dance

The EG participated in a psychomotor intervention mediated by creative dance (12 weeks; 3×/week; 60 min; 36 sessions). The sessions were designed and led by the study’s second author, who holds a degree in dance and psychomotricity.

The intervention aimed to enhance interoceptive body awareness (e.g., adjust breathing), spatial and temporal awareness (e.g., performing movements at different speeds and directions), and interrelationships (pairs and in groups) using diverse music styles. The intervention’s structured approach ensured a gradual progression in activity complexity (first 10 sessions: focusing on internal sensations of the body, such as breath or muscle tension; 11–20 sessions: emphasis on body awareness; 21–36 sessions: focusing on creativity and improvisation). The progressive structure of the sessions was intended to help participants move gradually from more guided body awareness to expressive, self-directed movement. By starting with basic awareness of body parts and moving toward rhythm and improvisation, the intervention aimed to support the internalization of bodily sensations and their integration with emotion and expression, promoting an integrated and embodied self-awareness. All sessions were performed at the gerontopsychomotricity laboratory. At the end of sessions, participants signed an attendance sheet and recorded their session participation.

Each session was structured as follows:

Initial dialog (5 min): Participants reviewed the previous session and were briefed on the current session’s activities.

Global activation (10 min): Tasks promoting physiological mobilization through body awareness activities.

Main phase (20 min): Participants created their movements to express ideas, emotions, and internal bodily sensations. They used images and objects such as scarves, balls, and chairs as facilitators of expression and communication. These props were not only helpful for encouraging movement variety, but also served to stimulate tactile and proprioceptive awareness. These sensations were meant to support participants’ ability to attend to bodily cues like breath, balance, or muscle tension, which are considered relevant to interoceptive awareness. Activities included breath dynamics (e.g., connecting breath with movement using quick, long, or deep breaths), muscle tension and relaxation dynamics, internal sensation exploration (e.g., associating body, emotions, and movements), heartbeat synchronization with calm/instrumental music, and balance and proprioception dynamics.

Choreographic phase (10 min): Participants recalled and created movements to be included in a final group choreography.

Cool down (10 min): Slower movements, breathing exercises, stretching, and relaxation.

Final dialog (5 min): Participants shared their experiences from the session.

Music was used as a mediator and was carefully selected to match each session phase according to the participants’ preferences, including various styles such as classical, instrumental, American, Brazilian, and traditional Portuguese music.

### 2.6 Statistical analysis

Data were analyzed using JASP software (version 0.18.3), with a significance level set at *p* < 0.05.

Based on the Shapiro–Wilk and Levene test results, the normality and homogeneity assumptions were not satisfied. Therefore, non-parametric statistical methods were used. The Wilcoxon and Mann–Whitney tests were used for within-group and between-group comparisons, respectively.

Means and standard deviations were used to present data. The change from baseline to post-intervention (variation) was calculated as ∆: moment_x_ – moment_x − 1_, and the respective delta percentage ∆%: [(moment_x_ – moment_x − 1_)/moment _x − 1_] × 100.

The clinical significance of the treatment effect for within-group or between-group comparisons was calculated following the guidelines for non-parametric tests (Fritz et al., 2012). The effect size was computed as *r* = (*Z*/√*N*), with Cohen’s thresholds for small (0.10), medium (0.30), and large (0.50) effects considered (Cohen, 1998).

## 3 Results

Participants presented similar sociodemographic characteristics at baseline, since no significant differences between groups were found in age (EG: 73.5 ± 5.9 years vs. CG: 75.7 ± 7.2 years), educational level (EG: 6.2 ± 2.9 years vs. CG: 5.3 ± 3.6 years), cognitive status (EG: 19.2 ± 0.4 points vs. CG: 19.2 ± 0.4 points), IMC (EG: 28.8 ± 3.4 years vs. CG: 27.5 ± 3.2 years), and gender (*p* > 0.05).

**Figure 1:**
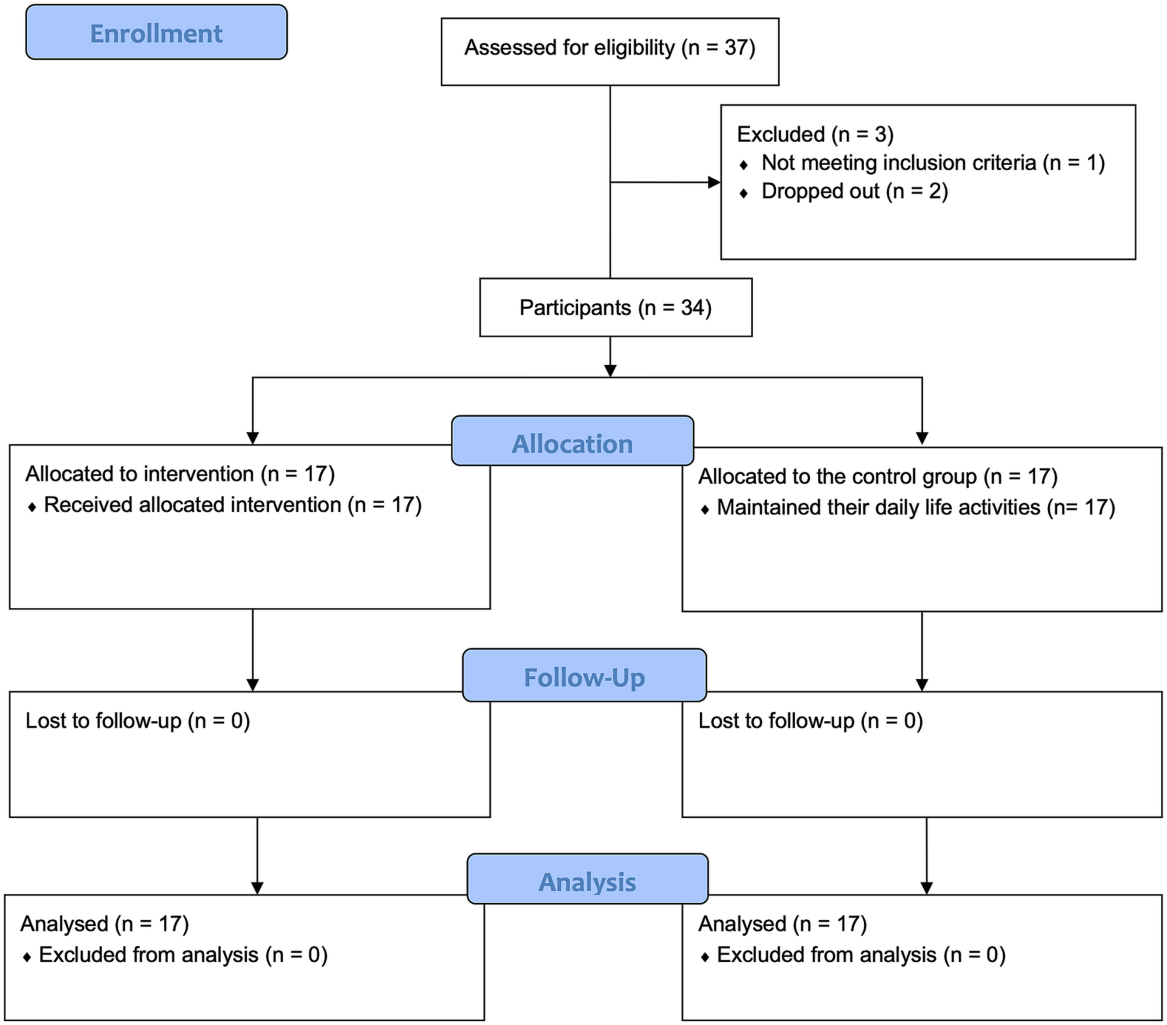
Flow diagram of the study participants.

Thirty-four participants completed this study. The attendance rate of the intervention (36 sessions) was 89.8%. No adverse events or falls were registered (Figure 1).

Table 1 presents the descriptive results for the MAIA scales. Both groups showed similar results at baseline, with no significant differences observed (*p* > 0.05). At *Δ* pre-post-intervention, significant differences between groups were noted in favor of the EG in all scales (*p* < 0.05), except for the Not-Distracting and Trusting.

**Table 1:** Descriptive results of the participant’s MAIA scales.

Variables	Baseline (mean ± SD)	Δ Post-intervention (mean ± SD)
Noticing (points)		
EG	9.4 ± 3.4	3.7 ± 3.3^a^
CG	9.8 ± 3.1	0.4 ± 1.2
Not-distracting (points)		
EG	3.4 ± 2.1	0.4 ± 3.1
CG	5.7 ± 4.1	−0.2 ± 1.9
Not-worrying (points)		
EG	10.4 ± 3.7	2.1 ± 3.4^a^
CG	11.5 ± 2.4	−0.2 ± 1.9
Attention regulation (points)		
EG	14.2 ± 5.2	8.2 ± 3.7^a^
CG	14.1 ± 5.7	1.4 ± 2.1
Emotional awareness (points)		
EG	16.1 ± 4.7	6.2 ± 4.0^a^
CG	17.6 ± 2.6	0.2 ± 2.9
Self-regulation (points)		
EG	17.1 ± 5.3	6.7 ± 4.6^a^
CG	16.7 ± 4.4	−1.5 ± 3.4
Trusting (points)		
EG	11.1 ± 2.2	1.6 ± 3.0
CG	10.9 ± 1.9	0.4 ± 0.9

SD, standard deviation; EG, experimental group (*n* = 17); CG, control group (*n* = 17).

a Significant differences between EG and CG, *p* < 0.05.

**Figure 2:**
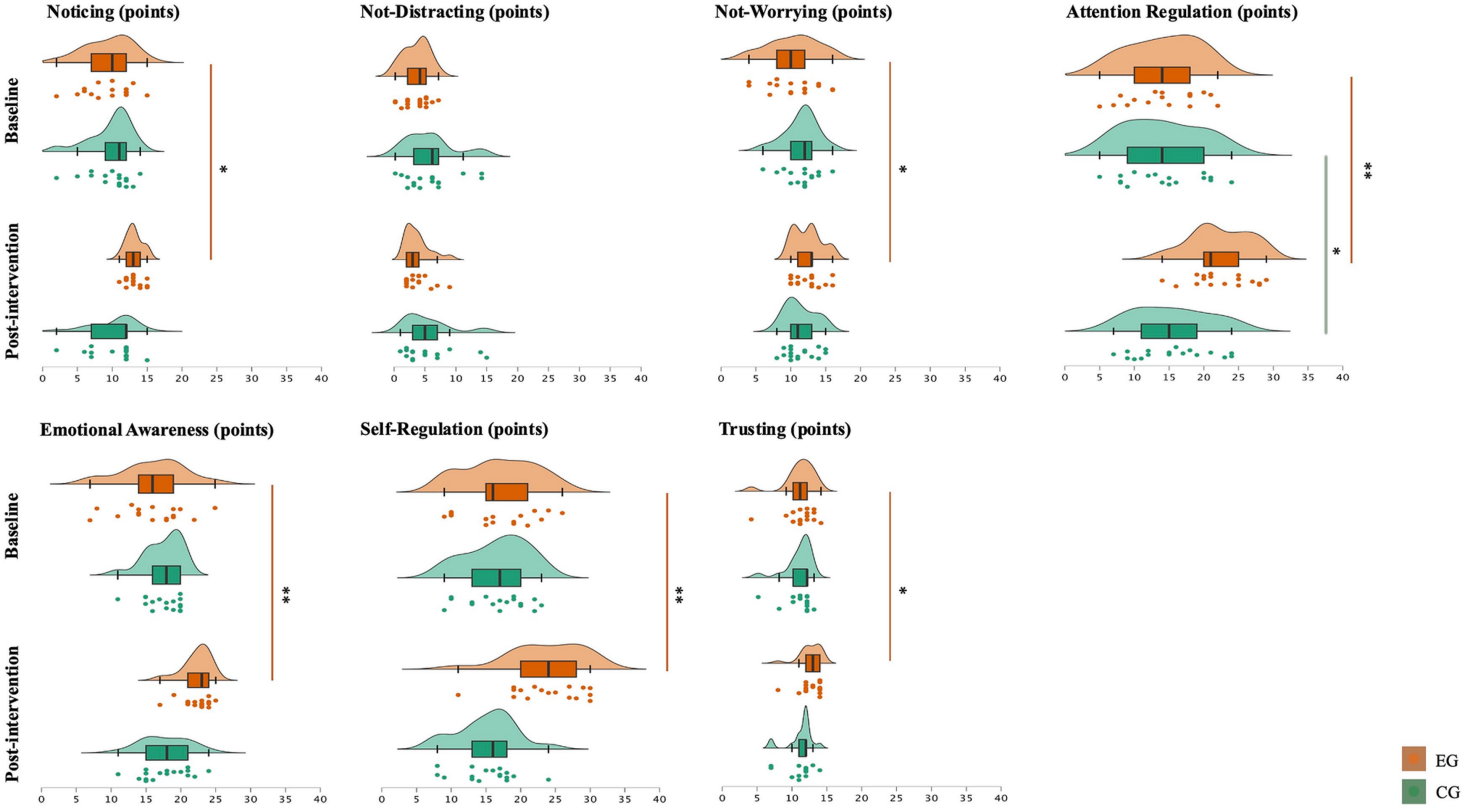
Density plots, box plots (median, interquartile range, minimum, and maximum), and jitter plots comparisons between the baseline and post-intervention evaluations; * significant differences within groups, *p* < 0.05; ** significant differences within groups, *p* < 0.001. EG: experimental group (*n* = 17); CG: control group (*n* = 17).

Figure 2 shows the differences within and between groups regarding the MAIA scales. Following 12 weeks of intervention, the results showed significant pre-post differences in the EG, with EG presenting significant enhancements in the scales of Noticing (∆%: 39.4, *p* = 0.002, *r* = 0.53), Not-Worrying (∆%: 19.9, *p* = 0.024, r = 0.39), Attention Regulation (∆%: 57.4, *p* < 0.001, *r* = 0.62), Emotional Awareness (∆%: 38.8, *p* < 0.001, *r* = 0.60), Self-Regulation (∆%: 39.3, *p* < 0.001, *r* = 0.59), and Trusting (∆%: 14.4, *p* = 0.026, *r* = 0.38). Overall, the CG maintained their results except for the scale Attention Regulation, which slightly increased (∆%: 9.6, *p* = 0.025, *r* = 0.38). The respective effect sizes ranged from medium to large.

Concerning differences between groups, the results were similar to those presented previously, highlighting an increase in the EG’s results. Significant differences were found after the intervention in the scales of Noticing (*p* < 0.001, *r* = 0.61), Attention Regulation (*p* < 0.001, *r* = 0.57), Emotional Awareness (*p* < 0.001, *r* = 0.64), Self-Regulation (*p* < 0.001, *r* = 0.72), and Trusting (*p* = 0.013, *r* = 0.45). The corresponding effect sizes ranged from medium to large.

## 4 Discussion

Our results suggest that a psychomotor intervention mediated by creative dance can effectively enhance interoceptive awareness in community-dwelling older adults. To our knowledge, this was the first study to explore such effects in this population. This is particularly relevant given the decline of interoceptive awareness with advancing age, which affects emotional experiences (Murphy et al., 2018; Ulus and Aisenberg-Shafran, 2022). Significant improvements were observed across all MAIA scales in the EG, except for Not-Distracting. These changes were clinically meaningful, with effect sizes ranging from medium to large. This suggests that creative dance, when integrated into a psychomotor intervention, may promote overall well-being in older adults by enhancing interoceptive and emotional self-regulation, thus contributing to healthy aging.

Between-group post-intervention comparisons reinforced the positive effects in the EG, with the CG showing stable or slightly declining results, except for a modest improvement in Attention Regulation. However, the effect size for this scale remained larger in the EG. This slight improvement in the CG might reflect increased self-awareness from study participation, particularly regarding attention-related sensations, which tend to remain stable over time (Tihanyi et al., 2017).

Adherence is often a barrier to the success of physical activity or exercise programs for older adults. Our intervention was considered acceptable, viable, and safe among older adults, with a completion rate of 89.8%, consistent with other creative dance programs (Fong Yan et al., 2018; Fong Yan et al., 2024). Factors such as the minimal requirement for equipment and the pleasant activities proposed in a social environment likely contributed to our higher adherence rate (Fong Yan et al., 2024).

Recent literature has suggested a positive impact of mindfulness practices on interoceptive and body awareness using the MAIA (Pérez-Peña et al., 2022). However, the limited number of studies investigating the effects of interventions involving creative dance and emphasizing body awareness in older adults (Cruz-Ferreira et al., 2015; Joung and Lee, 2019; Marmeleira et al., 2009) restricts comparisons and discussions with other studies, particularly on interoceptive awareness. Nevertheless, the previous studies found significant improvements in physical fitness, proprioception, and quality of life, highlighting the benefits of interventions involving creative dance focused on body awareness, which can contribute to effective emotional regulation (Ulus and Aisenberg-Shafran, 2022).

The observed increases in interoceptive awareness support prior evidence linking body awareness to psychological well-being (Hanley et al., 2017). A recent meta-analysis (Fong Yan et al., 2024) aligns with these results, suggesting that dance-based interventions may surpass other physical activities in enhancing emotional well-being by improving several psychological outcomes, such as physical awareness and self-regulation. Specifically, the enhancements observed in our study suggested that the EG participants achieved a greater awareness of bodily sensations in the capacity to notice (Noticing), not worry when faced with sensations of pain or discomfort (Not-Worrying), distress regulation (Self-Regulation), emotional states and attention regulation (Attention Regulation and Emotional Awareness), and the tendency to experience the body as safe, trustworthy, and predictable (Trusting). The study by Pérez-Pena and colleagues (Pérez-Peña et al., 2022) also showed enhancements in body awareness using MAIA, linking greater body awareness with more positive body sensations. However, they used a younger sample (mean age: 42.1 years) and a Mindfulness-Based Cognitive Therapy program, so this comparison should be interpreted with caution.

Interventions involving dance are associated with neuroplasticity, as the internal and external stimuli of dance can improve functional connectivity and increase brain volumes, promoting neuroplasticity in older adults (Nascimento, 2021). Engaging in dance tasks requires older adults to continuously adapt various neural and motor regions to the specific movements, choreographies, spatial orientations, and rhythms dictated by the music (Nascimento, 2021). Additionally, this process emphasizes the body’s central and active role in emotional and cognitive functioning, involving neural systems underlying movement-cognition and sensorimotor integration, perception, action, and emotion (Foster Vander Elst et al., 2023). This perspective helps explain how embodied and expressive interventions like creative dance can foster improvements in interoceptive awareness and emotional regulation.

Our study’s findings highlight the potential benefits of incorporating creative dance into psychomotor interventions within public health initiatives or community centers aimed at older adults. Given its holistic approach to enhancing physical, cognitive, and mental health (Fong Yan et al., 2018; Podolski et al., 2023; Predovan et al., 2019), interventions involving creative dance that integrate physical activity and emphasize body awareness can offer an effective strategy for promoting healthy aging.

The predominance of female participants in our sample may have influenced the outcomes, as women tend to report higher interoceptive sensibility (i.e., bodily sensations and emotional states) than men (Grabauskaitė et al., 2017). Moreover, women are generally more receptive to expressive and relational modalities such as dance, particularly in older age, which may foster higher engagement, enjoyment, and emotional openness during creative movement sessions. While the findings remain valid, caution is needed when generalizing them to older male populations, who may respond differently.

Future studies should include follow-up assessments—e.g., 12 weeks after program completion, matching the intervention duration—to examine the sustainability of effects on interoceptive awareness, as recent studies have also noted the lack of such data (Pérez-Peña et al., 2022). Additionally, combining self-reported and objective physiological measures (e.g., heartbeat tracking) to evaluate interoceptive awareness may offer a more comprehensive understanding of how participants perceive their internal states.

The present study has some strengths and limitations. Among the strengths, we highlight the innovative and engaging nature of the intervention and a high adherence rate. However, the study also has limitations. The non-randomized controlled trial design and relatively small sample size may limit the statistical power—particularly to detect small effects—and reduce the generalizability of the findings to broader populations. No statistical adjustments were applied to control for allocation bias, although baseline comparability between groups was ensured. Although the CDT accurately discriminates cognitively unimpaired individuals from those with early cognitive decline (Aprahamian et al., 2009), we recommend that future studies include a more comprehensive screening tool to assess cognitive performance. Furthermore, our study had a high prevalence of female participants, despite being consistent with another dance-based intervention (Fong Yan et al., 2024).

## 5 Conclusion

This study showed that a psychomotor intervention mediated by creative dance can significantly enhance interoceptive awareness in community-dwelling older adults. These improvements are particularly relevant considering the critical role of interoception in emotional regulation and overall well-being, especially in older adults. The clinical relevance of these findings, with effect sizes ranging from medium to large, supports the potential of this intervention as an effective, accessible, and enjoyable approach. Thus, by promoting physical activity and supporting both physical and mental health, this type of psychomotor intervention may offer a valuable strategy for healthy aging.

## Data Availability

The raw data supporting the conclusions of this article will be made available by the authors, without undue reservation.
